# Special Issue: Biogenic Nanomaterials: Versatility and Applications

**DOI:** 10.3390/molecules25092034

**Published:** 2020-04-27

**Authors:** Clayton Jeffryes, Si Amar Dahoumane

**Affiliations:** 1 Dan F. Smith Department of Chemical Engineering, Lamar University, Beaumont, TX 77710, USA; 2 School of Biological Sciences and Engineering, Yachay Tech University, Hacienda San José s/n, San Miguel de Urcuquí 100119, Ecuador

Greener processes have emerged as serious and competitive routes to produce various nanomaterials. These ecofriendly methodologies rely on the use of bioresources to both promote crystal growth and, very often, ensure the size and shape control and the colloidal stability of the as-fabricated nanomaterials, while concomitantly avoiding the use of chemicals and the generation of harmful by-products.

Nature provides materials scientists with valuable and renewable resources to typically carry out the synthesis of the desired nanomaterials at room or very mild temperatures, atmospheric pressure and aqueous solvents, therefore fulfilling the principles of Green Chemistry. So far, researchers have devised innovative methodologies to expand and advance the biosynthesis of unique nanomaterials by exploiting these bioresources. From a fundamental point-of-view, several research teams have shed light on the underlying synthesis mechanisms.

The present Special Issue of Molecules, titled *Biogenic Nanomaterials: Versatility and Applications*, covers several aspects of nanomaterial biosynthesis and their applications. For instance, Xiang et al. [1] extracted cellulose nanocrystals from cotton fibers, then successfully carried out their surface functionalization using cetyltrimethylammonium bromide (CTAB) to provide these widespread natural nanocrystals with interesting antifungal activity against *Phytophthora capsici*, a plant pathogen. These CTAB-functionalized cellulose nanocrystals proved to be an efficient and ecofriendly alternative to the use of chemical pesticides. On the other hand, Ghramh et al. [2] relied on the leaf extracts of *Euphorbia peplus* plants to promote the reduction of cationic gold into its metallic counterpart yielding the formation of gold nanoparticles (Au NPs) that exhibited several biological activities, such as the stimulatory effect on HeLa cell lines and an inhibitory effect on the HepG2 cancer cell line, in addition to larvicidal, bactericidal and fungicidal activity, among others.

Bacteria and their extracts have been intensively exploited by the community of material scientists. In their paper, Piacenza et al. [3] described the synthesis of metalloid NPs made of selenium by the bacterium *Ochrobactrum* sp. MPV1, studied the impact of selenate concentration and culturing conditions on the features of the as-produced Se NPs and proposed a hypothetical mechanism that could explain their outcomes. 

Magnetotactic Bacteria (MTB), a very diverse group of prokaryotes from metabolic and phylogenetic points-of-views, biomineralize magnetic nanoparticles via an intracellular process, giving rise to magnetosomes. The applications of these magnetic crystals, such in drug delivery, cell separation, hyperthermia treatment of cancer or as contrast agent in magnetic resonance imaging, are discussed in the review by Vargas et al. [4]. Inspired by biomineralization, Liu et al. [5] devised a methodology to produce superparamagnetic Fe_3_O_4_ nanocrystals via the smart combination of a first Fe_3_O_4_ binding peptide, with a second one consisting of a targeting ovarian cancer cell sequence and ginger extract. This yielded the production of a homogenous population of superparamagnetic Fe_3_O_4_ NPs of ~7 nm in size that are stable in both water and human serum. Moreover, these NPs exhibited great promise as contrasts agents in magnetic resonance imaging (MRI).

Using plant viruses, which are themselves nanoscale, as templates and delivering agents in nanotechnology is another novel way to exploit natural resources. Prominent findings in this field are summed up in the review by Zhang et al. [6]. Indeed, several plant viruses are described, and methods of their chemical modification and genetic engineering are highlighted, along with their self-assembly; finally, their various bioapplications are detailed, such as bioimaging, therapeutic delivery and nanobioreactor design, among others.

In their trilogy, Rahman et al. compared the impact of the presence and absence of the cell wall in the green, unicellular microalga *Chlamydomonas reinhardtii* on the features of the biosynthesized silver nanoparticles mediated by this photosynthetic microorganism in terms of shape, size, colloidal stability, synthesis kinetics and conversion yield [7]. Their second paper depicts a comparison between the use of whole living cultures of the same microalgal species, its washed cells and supernatant on the same features [8]. Their last paper focuses on the comprehension of the mechanism of Ag NP photocatalytic production by the extracellular polymeric substances of *C. reinhardtii* [9].

In summary, the readership will discover, in the present Special Issue, prominent and diverse facets of bionanotechnology and nanobiotechnology spanning from the synthesis of inorganic nanomaterials using natural bioresources, to the advantages of natural nanostructures, including their bioapplications.
